# A systematic scoping review on quality criteria for clinical practice guidelines for rare diseases

**DOI:** 10.1186/s13643-026-03247-1

**Published:** 2026-07-16

**Authors:** Iméze J. Hieltjes, Mirthe J. Klein Haneveld, Ingeborg Van Dusseldorp, Charlotte M. W. Gaasterland, Johanna H. van der Lee

**Affiliations:** 1 Knowledge Institute of the Dutch Association of Medical Specialists, Utrecht, The Netherlands; 2 Amsterdam UMC, University of Amsterdam, Emma Children’s Hospital, Amsterdam, The Netherlands; 3 European Reference Network On Rare Congenital Malformations and Rare Intellectual Disability ERN-ITHACA, Clinical Genetics Department, Robert Debré University Hospital, Paris, France; 4 Amsterdam Reproduction and Development Research Institute, Amsterdam, The Netherlands; 5 Amsterdam Public Health Research Institute, Amsterdam, The Netherlands; 6 Department of Epidemiology, Leiden University Medical Center, Leiden, The Netherlands

## Abstract

**Background:**

Rare diseases impose substantial challenges on affected individuals and healthcare systems. While clinical practice guidelines (CPGs) are crucial for standardizing care, tools to assess their trustworthiness are limited, particularly for rare diseases. There is a need for minimum quality criteria to ensure CPG reliability and utility.

**Objective:**

This systematic scoping review aims to identify a set of criteria that can be used for evaluating and endorsing CPGs for rare diseases.

**Methods:**

A systematic scoping review was conducted using four databases (Ovid/Medline, Embase.com, Scopus, and Google Scholar) from inception to April 9, 2024. The search was developed by a medical information specialist. Titles and abstracts were independently screened by two reviewers, with disagreements resolved through discussion or a third reviewer. Articles were included if they addressed quality criteria for guidelines on rare diseases in general, excluding disease-specific guidelines.

**Results:**

From 9587 unique titles, only one study met the inclusion criteria. The included study, published by Hilton-Boon et al. (2015), summarized an international workshop on the applicability of AGREE II criteria for rare disease guidelines.

**Conclusion:**

Our systematic review identified a single report detailing an international workshop that evaluated the utility of the AGREE II instrument for assessing two guidelines focused on rare diseases. This limited finding highlights a significant gap in methodologies tailored to the specific complexities of rare disease guidelines. The findings underscore the need for a tailored, streamlined set of criteria to address the unique challenges of rare disease guidelines, supporting their development, evaluation, and endorsement in clinical practice.

**Supplementary Information:**

The online version contains supplementary material available at https://doi.org/10.1186/s13643-026-03247-1.

## What is new?

Key findings

- Despite a sensitive search in this systematic review, we did not find a set of quality criteria for the specific context of rare disease guidelines. We only identified a single report describing an international workshop that examined the applicability of the AGREE II instrument for evaluating two guidelines for one rare disease.

What this adds to what is known?

- This shows the significant lack of quality criteria tailored to the specific complexities of rare disease guidelines.

What is the implication and what should change now?

- Guidelines for rare diseases may benefit from a tailored, streamlined set of criteria to address the unique challenges of the rare disease field, supporting the development, evaluation, and endorsement of these guidelines.

## Introduction

In Europe, a disease is considered rare if it affects no more than 1 in 2000 persons [1, 2]. Around six to seven thousand rare diseases have been discovered, and new diseases are identified regularly [3]. Although rare diseases individually affect a small number of people, collectively they have a considerable impact on the population [1]. Due to limited knowledge and less robust evidence on diagnosis and interventions, the quality of care may be lower compared to more prevalent diseases [4]. In 2009, the European Commission recommended establishing and implementing national plans and strategies to ensure that patients with rare diseases have access to high-quality care [5].

Clinical practice guidelines (CPGs) are systematically developed statements or recommendations that assist healthcare providers in making informed decisions about the care of patients. These CPGs are based on a thorough review of the best available evidence from clinical research and expert opinion (including both clinical and patient expertise). They aim to standardize and improve patient care by providing clear, evidence-based recommendations for diagnosing, managing, and treating specific medical conditions or diseases [6]. Additionally, CPGs serve as a valuable resource for ensuring consistency in healthcare, reducing practice variability, and supporting shared decision-making between patients and providers. However, developing CPGs for rare diseases poses several challenges [7–12].

First, it is difficult to decide which topics to address in a rare disease CPG given the limited resources and evidence. For many rare diseases, there are still considerable knowledge gaps with regard to the best way to diagnose, manage, and treat patients. When resources are scarce, it is challenging to decide for which rare disease a CPG should be developed and which clinical questions for this disease need to be addressed most urgently.

Another challenge is that the existing literature on rare diseases often consists of case studies or non-comparative observational designs, resulting in very low certainty of evidence. Due to limited resources, guideline development panels may refrain from performing a systematic review since they do not expect considerable high-level evidence, and in this way make methodological shortcuts [13–15]. Also, guideline development panels in rare diseases are not always aware of existing methodologies for guideline development in our experience as methodologists.

Not only the number of patients is low in rare diseases; also, the number of medical professionals and patient representatives may be limited, which may lead to insufficient representation of stakeholders when developing a CPG. In a small group, the influence of an individual (either patient expert or clinical expert) may be out of proportion and may not always align with the interests of all stakeholders. Personal differences are of more influence in small expert groups. This issue is less salient in non-rare diseases, where there is a larger group of stakeholders to choose from.

Finally, related to the former issue, it is not always possible to maintain editorial independence in the development of rare diseases CPGs. The limited number of experts in rare diseases makes it particularly difficult to find guideline panel members without competing interests.

Due to these challenges, not all documents that are called guidelines or CPGs for rare diseases are developed according to the standard methodology [10]. However, clinicians still use such documents to make informed decisions on the management and treatment of their patients. Therefore, clinicians are helped when they know how to distinguish trustworthy from untrustworthy CPGs [16]. Lima et al. 2023 [16] give guidance to clinicians for the distinction of trustworthy and untrustworthy guidelines in general, not limited to rare diseases. However, the eight domains they propose as a conceptual framework for evaluating the trustworthiness of guidelines are intended as a flexible guide rather than a set of criteria.

Endorsement by official organizations such as European Reference Networks (ERNs) or medical specialist scientific associations may be instrumental to distinguish trustworthy documents from less trustworthy documents. These organizations need ways to appraise the quality of CPGs or other quality documents for rare diseases that are offered to them for endorsement and official publication on the organization’s website. The AGREE II instrument was designed to assess the methodological quality and transparency of CPGs [17]. It contains 23 items in six domains. Several studies that have used the AGREE II instrument to appraise CPGs for rare diseases [7–12] show considerable variation in quality and highlight the lack of methodological rigor of CPGs for rare diseases. While the AGREE II checklist represents a comprehensive standard, achieving high scores on all 23 quality items has its challenges for CPGs for rare diseases as shown before. Therefore, the development and appraisal of CPGs for rare diseases may need a specific set of criteria for rare disease CPGs, to enhance the feasibility of developing and endorsing such documents in the context of rare diseases. These criteria should ensure that CPGs meet essential quality benchmarks without necessitating perfection, thus providing a minimum level of reliability and utility for healthcare providers of rare diseases.

This systematic scoping review aims to identify a set of criteria that can be used for evaluating and endorsing CPGs for rare diseases.

## Methods

For this scoping review, a systematic sensitive literature search was performed to identify as many relevant articles as possible [18]. The search strategy was set up by a medical information specialist (I.v.D.) of the Knowledge Institute of the Dutch Association of Medical Specialists, using the following bibliographic databases: Ovid/Medline, Embase.com, Scopus and Google Scholar. The literature was searched from inception until 9-4-2024. The systematic search was completed using a combination of controlled vocabulary wherever available, and natural language title, abstract and keywords. The overall search strategy was derived from three primary search concepts: “rare diseases” and “quality criteria” and “guidelines/standards.” Duplicates were removed using EndNote software. The detailed search strategy can be found in Appendix 1.

### Study selection

Titles and abstracts were independently screened by two reviewers (I.H. and M.K.). If considered potentially relevant, the full texts were independently assessed by two authors (I.H. and M.K).

Disagreements between reviewers were discussed until consensus. If necessary, a third reviewer (C.G.) was consulted. The following inclusion criteria were used:

1. Articles about rare diseases in general and not about a specific disease
2. Articles presenting quality criteria for guidelines, standards or quality documents for rare diseases

The exclusion criteria were:

1. Articles in other languages than English or Dutch
2. Posters or conference abstracts
3. Guidelines for specific (rare) diseases
4. Articles about quality documents for a non-rare disease

### Data analysis plan

We planned to use the information in the included articles to make an overview of all available sets of criteria and how these criteria were developed. We intended to compare different sets of criteria to identify criteria that were common to all sets of criteria, and to critically assess criteria that were not included in more than one set of criteria.

## Results

**Fig. 1:**
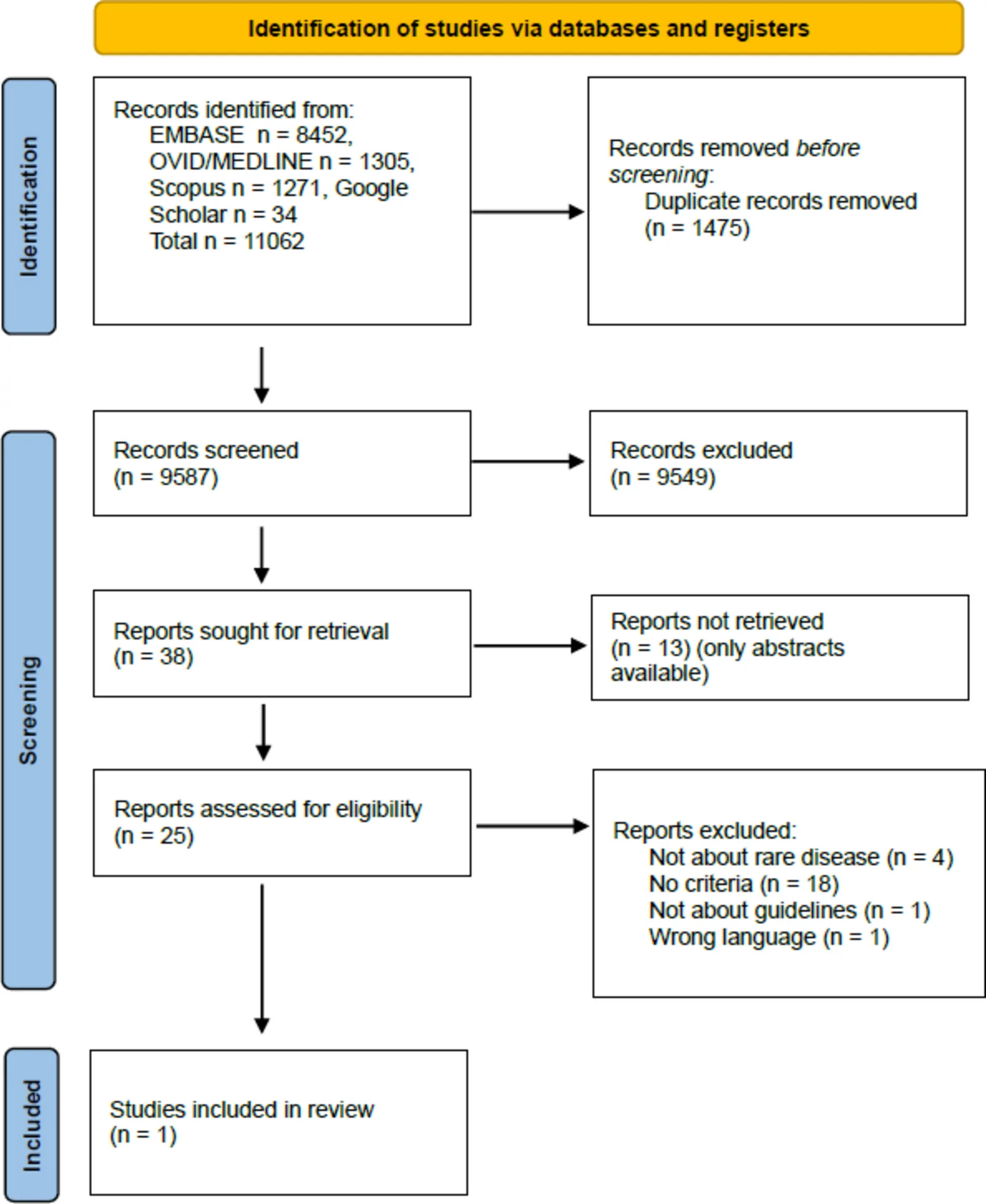
Prisma flow chart

Our comprehensive search yielded 9587 unique titles. Thirty-eight full-text articles were assessed, but only one article met the inclusion criteria (Fig. 1). Most articles were excluded during the title and abstract screening because they did not report quality criteria or because they concerned a specific rare disease. We found several articles that were guidelines for developing CPGs for rare diseases [19, 20].

The one article that met our inclusion criteria was published by Hilton-Boon et al. (2015) , reporting on an international workshop aimed at evaluating the applicability of the AGREE II criteria for appraising CPGs related to rare diseases. During this workshop, participants completed the AGREE II online tutorial and then worked in small groups to assess two guidelines about Huntington’s disease. The two guidelines had been selected because they “provided sufficient information to support informed judgment on most if not all AGREE items” [21]. Therefore, it was not surprising that the attendees concluded that the AGREE II instrument could be used to appraise rare disease CPGs. Based on the discussions about the challenges and disagreements encountered when applying AGREE II to these two guidelines, several points to consider when using the instrument for the appraisal of rare disease CPGs were proposed as an addition to the manual of the AGREE II instrument.

## Discussion

This systematic scoping review aimed to identify a minimum set of criteria that can be used for evaluating and endorsing CPGs for rare diseases.

The extensive review only revealed one report concluding that the AGREE II [17] criteria could be employed to evaluate the quality of CPGs for rare diseases according to an international workshop with members of the RARE best practices consortium [21]. The report of this workshop by Hilton Boon et al. (2015) proposed additions to the AGREE II manual for the appraisal of CPGs for rare diseases. Despite a thorough search, no other set of criteria beyond AGREE II was identified. While the AGREE II checklist offers a comprehensive and widely recognized standard, achieving high scores across all 23 quality items presents significant challenges, particularly for CPGs addressing rare diseases. These challenges are related to the uncertainty of evidence, limited resources, insufficient representation of stakeholders, lack of editorial independence, and lack of awareness of existing guideline development methodology in rare disease experts, as argued in the introduction.

One of the strengths of our systematic scoping review was the extensive and rigorous search strategy, with well-defined inclusion and exclusion criteria. This approach ensured a thorough examination of the existing literature on the topic. However, only a single article met our criteria, suggesting a significant gap in the available research. Variations in the definitions of guidelines or quality documents related to rare diseases may have led to the failure to discover relevant articles that used alternative but conceptually similar terminology; furthermore, restricting our search to English-language publications may have introduced a language bias, potentially leading to the omission of relevant studies published in other languages.

However, we are convinced that if a set of criteria specifically aimed to appraise CPGs for rare diseases were to exist, this sensitive search would have found it.

This review has several limitations. First, grey literature was not included in the search strategy. Consequently, relevant ongoing work or findings reported in non-peer-reviewed sources, such as conference abstracts or institutional reports, may have been missed. However, the search strategy was quite sensitive, as is shown in the large number of articles screened compared to the number of articles included. Therefore, we are confident that no major insights or widely used criteria set was missed, and that we may conclude that such a criteria set is currently missing.

Second, the search was not extended to include disease-specific studies. Although this might have yielded additional criteria, such criteria are likely to be context-dependent and therefore less applicable across rare diseases. Given that this review aimed to identify overarching quality criteria to support the endorsement of rare disease guidance documents, we intentionally focused on literature addressing rare diseases in general.

Based on these results, the AGREE II instrument is the only set of criteria that is recommended to evaluate the quality of rare disease CPGs, supplemented by the guidance provided by Hilton Boon et al., 2015 [21]. However, since the AGREE II set may be overly ambitious in the context of rare diseases, we strongly recommend the development of a set of criteria that is more tailored to the context of rare diseases.

## Conclusion

We did not find a set of criteria specifically designed for evaluating and endorsing CPGs for rare diseases. Our comprehensive literature search yielded one report of a workshop in which international experts discussed the utility of the AGREE II instrument for the appraisal of two guidelines about one rare disease. AGREE II may be overly ambitious to evaluate CPGs in the context of rare diseases; this highlights the need to develop a leaner set of criteria tailored to the specific challenges of rare diseases, which will better support the evaluation and endorsement of CPGs in this field.

## Supplementary Information

Supplementary Material 1. Appendix 1.

## Data Availability

Data sharing not applicable to this article as no datasets were generated or analyzed during the current study.
