# Supplementary material for: A systematic scoping review on quality criteria for clinical practice guidelines for rare diseases
Source: Syst Rev. 2026 Jul 16;15:240. doi: 10.1186/s13643-026-03247-1 (PMC13540827; doi:10.1186/s13643-026-03247-1)
Supplement: Supplementary file 1 [file 13643_2026_3247_MOESM1_ESM.docx]

# Appendix 1

***Search strategy***

| Scoping review | |
| --- | --- |
|  | |
| Databases: Ovid/Medline, Embase | Date: 6-5-2021, 8-9-2022, 28-9-2022, 9-11-2022 |
| Medical information specialist: Ingeborg van Dusseldorp |  |
|  | |
| 1. *Rare diseases*   Rare diseases  'rare disease'/de OR (((rare OR unsusal OR orphan) NEAR/3 (disorder* OR disease* OR defect* OR anomali* OR syndrome* OR condition*)):ti,ab,kw)  Because a systematic review is being conducted, the decision was made to omit the major term and instead opt for the broader general keyword "rare disease." By using /de, all references with this keyword are displayed, not just those where it is the primary focus.   2. *Quality criteria*   'quality control procedures'/de OR 'certified reference material'/exp OR 'quality assessment tool'/exp OR 'quality control'/exp OR 'reference measurement procedure'/exp OR 'standardization'/exp OR 'appraisal'/de OR appraisal:ti,ab,kw OR assessment:ti,ab,kw OR evaluation:ti,ab,kw OR ((quality NEAR/3 (control OR tool* OR methodolog* OR instrument*)):ti,ab,kw)  Standardization has been added as agreed, but we also opted for "quality control procedures" instead of just "quality control." Since using "quality control procedures" generated a lot of irrelevant results, certain terms, such as "monitoring," were excluded. The keywords that were included are highlighted in yellow (except for "standardization"). The terminology has not yet been fully elaborated. For example, subterms for "quality assessment tools" have not yet been defined in titles, abstracts, or keywords. After the terminology is approved, further refinement can be undertaken.   3. *Guidelines, standards*   'practice guideline'/exp OR 'quality document*':ti,ab,kw OR guideline*:ti,ab,kw OR standard*:kw OR ((consensus NEAR/3 (development OR document*)):ti,ab,kw) OR ((guide NEAR/4 (clinical OR evidence OR 'best practice' OR bestpractice)):ti,ab,kw) OR 'standard of care':ti,ab,kw  It was decided to include "standard*" only as an author keyword to avoid excessive irrelevant results related to standard care, standard medication, etc. However, "standard of care" is included. | |

***Search results***

| 9-4-2024 | **EMBASE** | **OVID/MEDLINE** | **Scopus** | **Deduplicates Rayyan 9-11-2022** |
| --- | --- | --- | --- | --- |
| SR | 1073 | 277 |  | 539 |
| RCT |  |  |  |  |
| Observational studies |  |  |  |  |
| Overig | 7379 | 1028 |  | 4008 |

***Search strategies***

**Embase**

**9-11-2022**

| No. | Query | Results |
| --- | --- | --- |
| #30 | #25 NOT #29 overige | 3389 |
| #29 | #25 AND #28 SR | 535 |
| #28 | 'meta analysis'/exp OR 'meta analysis (topic)'/exp OR metaanaly*:ti,ab OR 'meta analy*':ti,ab OR metanaly*:ti,ab OR 'systematic review'/de OR 'cochrane database of systematic reviews'/jt OR prisma:ti,ab OR prospero:ti,ab OR (((systemati* OR scoping OR umbrella OR 'structured literature') NEAR/3 (review* OR overview*)):ti,ab) OR ((systemic* NEAR/1 review*):ti,ab) OR (((systemati* OR literature OR database* OR 'data base*') NEAR/10 search*):ti,ab) OR (((structured OR comprehensive* OR systemic*) NEAR/3 search*):ti,ab) OR (((literature NEAR/3 review*):ti,ab) AND (search*:ti,ab OR database*:ti,ab OR 'data base*':ti,ab)) OR (('data extraction':ti,ab OR 'data source*':ti,ab) AND 'study selection':ti,ab) OR ('search strategy':ti,ab AND 'selection criteria':ti,ab) OR ('data source*':ti,ab AND 'data synthesis':ti,ab) OR medline:ab OR pubmed:ab OR embase:ab OR cochrane:ab OR (((critical OR rapid) NEAR/2 (review* OR overview* OR synthes*)):ti) OR ((((critical* OR rapid*) NEAR/3 (review* OR overview* OR synthes*)):ab) AND (search*:ab OR database*:ab OR 'data base*':ab)) OR metasynthes*:ti,ab OR 'meta synthes*':ti,ab | 872392 |
| #27 | #21 NOT #26 | 2 |
| #26 | #21 AND #25 sleutelartikelen gevonden | 23 |
| #25 | #22 AND #23 AND #24 | 3918 |
| #24 | 'practice guideline'/exp OR 'standard'/exp OR 'quality document*':ti,ab,kw OR guideline*:ti,ab,kw OR standard*:kw OR ((consensus NEAR/3 (development OR document*)):ti,ab,kw) OR ((guid* NEAR/4 (clinical OR evidence OR 'best practice' OR bestpractice)):ti,ab,kw) OR 'standard of care':ti,ab,kw | 1579515 |
| #23 | 'quality control procedures'/de OR 'certified reference material'/exp OR 'quality assessment tool'/exp OR 'quality control'/exp OR 'reference measurement procedure'/exp OR 'standardization'/exp OR 'appraisal'/de OR apprais*:ti,ab,kw OR assess*:ti,ab,kw OR evaluati*:ti,ab,kw OR ((quality NEAR/3 (control OR tool* OR methodolog* OR instrument*)):ti,ab,kw) | 7662289 |
| #22 | 'rare disease'/de OR (((rare OR unusual OR orphan*) NEAR/3 (disorder* OR disease* OR defect* OR anomal* OR syndrome* OR condition*)):ti,ab,kw) | 246166 |
| #21 | #1 OR #2 OR #3 OR #4 OR #5 OR #6 OR #7 OR #8 OR #9 OR #10 OR #11 OR #12 OR #13 OR #14 OR #15 OR #16 OR #17 OR #18 OR #19 OR #20 sleutelartikelen | 25 |
| #20 | methodology AND in AND clinical AND a AND report AND the AND american AND college AND of AND heart AND association AND performance AND measures AND task AND force AND on AND practice AND guidelines AND anderson | 3 |
| #19 | of AND myasthenia AND centres AND developing AND implementing AND a AND certification AND procedure AND for AND patient AND organisations | 1 |
| #18 | review AND evaluation AND methodological AND quality AND of AND the AND existing AND guidelines AND recommendations AND for AND inherited AND neurometabolic AND disorders | 2 |
| #17 | treatment AND guidelines AND for AND five AND rare AND neurodevelopmental AND disorders AND a AND targeted AND literature AND review | 1 |
| #16 | pns148 AND differences AND similarities AND the AND level AND of AND evidentiary AND requirements AND required AND for AND orphan AND drugs AND during AND hta AND appraisal AND in AND cee AND countries | 1 |
| #15 | interface AND management AND concepts AND healthcare AND rare AND diseases AND in AND germany AND protocol AND for AND a AND 'mixed methods' AND study AND to AND develop AND best AND practice AND recommendations | 1 |
| #14 | how AND reference AND networks AND develop, AND implement, AND monitor AND guidelines | 1 |
| #13 | guideline AND for AND diagnosis AND management AND of AND catastrophic AND antiphospholipid AND syndrome AND legault AND 2016 | 2 |
| #12 | clinical AND practice AND guidelines AND in AND rare AND diseases AND leone AND 2017 | 1 |
| #11 | developing AND methodology AND the AND creation AND of AND clinical AND practice AND guidelines AND for AND rare AND diseases AND a AND report AND from AND 'rare bestpractices' | 1 |
| #10 | strategies AND eliciting AND synthesizing AND evidence AND for AND guidelines AND in AND rare AND diseases AND 2019 | 1 |
| #9 | clinical AND practice AND guidelines AND for AND rare AND diseases AND the AND orphanet AND database AND 2017 AND pavan | 1 |
| #8 | towards AND perfect AND arm AND center AND european AND criteria AND for AND centers AND of AND expertise AND their AND implementation AND in AND member AND states. AND a AND report AND from AND the AND 'arm net' | 1 |
| #7 | methodology AND production AND of AND best AND practice AND guidelines AND for AND rare AND diseases | 1 |
| #6 | patient AND journeys AND personal AND experiences AND shaping AND clinical AND priorities NOT 10th:ti | 1 |
| #5 | for AND uveal AND melanoma AND a AND critical AND appraisal AND of AND systematically AND identified AND guidelines AND using AND the AND agree AND ii AND 'agree rex' AND instrument | 1 |
| #4 | centres AND expertise AND european AND reference AND networks AND key AND issues AND in AND field AND of AND rare AND diseases. AND the AND eucerd AND recommendations | 1 |
| #3 | 'rare bestpractices' AND a AND platform AND sharing AND best AND practices AND for AND the AND management AND of AND rare AND diseases AND taruscio AND 2014 | 2 |
| #2 | developing AND hta AND guidelines AND for AND rare AND disease AND 'therapies an' AND industry AND perspective | 1 |
| #1 | 'decision making' AND rare AND diseases AND developing AND 'evidence based' AND clinical AND practice AND guidelines AND for AND growth AND hormone AND therapy AND in AND 'prader willi' AND syndrome AND using AND the AND evidem AND framework | 1 |

**Ovid/Medline**

**9-11-2022**

| **#** | **Searches** | **Results** |
| --- | --- | --- |
| 7 | 4 not 6 overige | 840 |
| 6 | 4 and 5 SR | 202 |
| 5 | (meta-analysis/ or meta-analysis as topic/ or (metaanaly* or meta-analy* or metanaly*).ti,ab,kf. or systematic review/ or cochrane.jw. or (prisma or prospero).ti,ab,kf. or ((systemati* or scoping or umbrella or "structured literature") adj3 (review* or overview*)).ti,ab,kf. or (systemic* adj1 review*).ti,ab,kf. or ((systemati* or literature or database* or data-base*) adj10 search*).ti,ab,kf. or ((structured or comprehensive* or systemic*) adj3 search*).ti,ab,kf. or ((literature adj3 review*) and (search* or database* or data-base*)).ti,ab,kf. or (("data extraction" or "data source*") and "study selection").ti,ab,kf. or ("search strategy" and "selection criteria").ti,ab,kf. or ("data source*" and "data synthesis").ti,ab,kf. or (medline or pubmed or embase or cochrane).ab. or ((critical or rapid) adj2 (review* or overview* or synthes*)).ti. or (((critical* or rapid*) adj3 (review* or overview* or synthes*)) and (search* or database* or data-base*)).ab. or (metasynthes* or meta-synthes*).ti,ab,kf.) not (comment/ or editorial/ or letter/ or ((exp animals/ or exp models, animal/) not humans/)) | 599833 |
| 4 | 1 and 2 and 3 | 1042 |
| 3 | exp Guideline/ or quality document*.ti,ab,kf. or guideline*.ti,ab,kf. or standard*.ti,kf. or (consensus adj3 (development or document*)).ti,ab,kf. or (guid* adj4 (clinical or evidence or best practice or bestpractice)).ti,ab,kf. or standard of care.ti,ab,kf. | 655192 |
| 2 | exp quality control/ or apprais*.ti,ab,kf. or assess*.ti,ab,kf. or evaluati*.ti,ab,kf. or (quality adj3 (control or tool* or methodolog* or instrument*)).ti,ab,kf. | 4972320 |
| 1 | exp Rare Diseases/ or ((rare or unusual or orphan*) adj3 (disorder* or disease* or defect* or anomal* or syndrome* or condition*)).ti,ab,kf. | 157958 |

**Google Scholar**

**"rare disease*" grade**

**"rare disease*" "quality assessment" guideline***

**Scopus**

**9-11-2022**

1

[Edit](https://www-scopus-com.proxy-ub.rug.nl/search/history/edit.uri?shid=11)

( TITLE-ABS-KEY ( ( "rare disease*" ) OR ( ( rare OR unusual OR orphan* ) W/3 ( disorder* OR disease* OR defect* OR anomal* OR syndrome* OR condition* ) ) ) ) AND ( TITLE-ABS-KEY ( quality AND control OR apprais* OR assess* OR evaluati* OR ( quality W/3 ( control OR tool* OR methodolog* OR instrument* ) ) ) ) AND ( ( TITLE-ABS-KEY ( quality AND document* OR guideline* OR standard* ) ) OR ( TITLE-ABS-KEY ( ( consensus W/3 ( development OR document* ) ) ) ) OR ( TITLE-ABS-KEY ( ( guid* W/4 ( clinical OR evidence OR bestpractice ) ) OR "standard of care" ) ) ) AND ( LIMIT-TO ( DOCTYPE , "re" ) )= SR

[373](https://www-scopus-com.proxy-ub.rug.nl/search/history/results.uri?origin=searchhistory&shid=11) results

2

[Edit](https://www-scopus-com.proxy-ub.rug.nl/search/history/edit.uri?shid=10)

( TITLE-ABS-KEY ( ( "rare disease*" ) OR ( ( rare OR unusual OR orphan* ) W/3 ( disorder* OR disease* OR defect* OR anomal* OR syndrome* OR condition* ) ) ) ) AND ( TITLE-ABS-KEY ( quality AND control OR apprais* OR assess* OR evaluati* OR ( quality W/3 ( control OR tool* OR methodolog* OR instrument* ) ) ) ) AND ( ( TITLE-ABS-KEY ( quality AND document* OR guideline* OR standard* ) ) OR ( TITLE-ABS-KEY ( ( consensus W/3 ( development OR document* ) ) ) ) OR ( TITLE-ABS-KEY ( ( guid* W/4 ( clinical OR evidence OR bestpractice ) ) OR "standard of care" ) ) )

[1271](https://www-scopus-com.proxy-ub.rug.nl/search/history/results.uri?origin=searchhistory&shid=10) results
